# Specific β-Tubulin Isotypes Can Functionally Enhance or Diminish Epothilone B Sensitivity in Non-Small Cell Lung Cancer Cells

**DOI:** 10.1371/journal.pone.0021717

**Published:** 2011-06-29

**Authors:** Pei Pei Gan, Joshua A. McCarroll, Frances L. Byrne, James Garner, Maria Kavallaris

**Affiliations:** 1 Children's Cancer Institute Australia, Lowy Cancer Research Centre, University of New South Wales, Randwick, Australia; 2 School of Chemistry, University of New South Wales, Sydney, Australia; 3 Australian Centre for Nanomedicine, University of New South Wales, Sydney, Australia; Weill Cornell Medical College of Cornell University, United States of America

## Abstract

Epothilones are a new class of microtubule stabilizing agents with promising preclinical and clinical activity. Their cellular target is β-tubulin and factors influencing intrinsic sensitivity to epothilones are not well understood. In this study, the functional significance of specific β-tubulin isotypes in intrinsic sensitivity to epothilone B was investigated using siRNA gene knockdown against βII-, βIII- or βIVb-tubulins in two independent non-small cell lung cancer (NSCLC) cell lines, NCI-H460 and Calu-6. Drug-treated clonogenic assays showed that sensitivity to epothilone B was not altered following knockdown of βII-tubulin in both NSCLC cell lines. In contrast, knockdown of βIII-tubulin significantly increased sensitivity to epothilone B. Interestingly, βIVb-tubulin knockdowns were significantly less sensitive to epothilone B, compared to mock- and control siRNA cells. Cell cycle analysis of βIII-tubulin knockdown cells showed a higher percentage of cell death with epothilone B concentrations as low as 0.5 nM. In contrast, βIVb-tubulin knockdown cells displayed a decrease in epothilone B-induced G_2_-M cell cycle accumulation compared to control siRNA cells. Importantly, βIII-tubulin knockdowns displayed a significant dose-dependent increase in the percentage of apoptotic cells upon treatment with epothilone B, as detected using caspase 3/7 activity and Annexin-V staining. Higher concentrations of epothilone B were required to induce apoptosis in the βIVb-tubulin knockdowns compared to control siRNA, highlighting a potential mechanism underlying decreased sensitivity to this agent. This study demonstrates that specific β-tubulin isotypes can influence sensitivity to epothilone B and may influence differential sensitivity to this promising new agent.

## Introduction

The taxanes (including paclitaxel and docetaxel) are established drugs widely used in the treatment of several types of solid tumours, including ovarian, breast, lung and head and neck cancer, either singly or in combination with other chemotherapeutic agents. The clinical success of taxanes has provided the impetus to search for other new agents with similar properties but with improved efficacy. Epothilones are a novel class of non-taxane microtubule-stabilizing agents that have shown promising anticancer activity. Among them, the epothilone B analogue, Ixabepilone (BMS-247550, aza-EpoB) was approved in 2007 by the Food and Drug Administration for the treatment of metastatic or locally advanced breast cancer resistant to anthracyclines, taxanes and capecitabine, either singly or in combination with these agents [1]. The naturally occurring epothilone B (patupilone, EPO906), has also shown promising activity in various preclinical models that are resistant to taxane-based chemotherapy and is currently under phase II/III clinical trials [2], [3], [4], [5]. Despite little structural similarity between the epothilones and the taxanes, both agents share the same or an overlapping binding site on β-tubulin [6], [7]. Similar to taxanes, epothilones induce microtubule bundling [6], suppress microtubule dynamics; leading to inhibition of cell proliferation and mitotic block [8]. Although epothilones and taxanes stabilize microtubules against depolymerization, they exhibit distinct differences in activity and efficacy (reviewed in [9], [10]).

Both epothilones and taxanes can stabilize microtubules against depolymerization, yet they exhibit distinct differences in activity and efficacy (reviewed in [9], [10]). The reasons for differences in activity are poorly understood. To date, studies have focused on acquired resistance to epothilones using drug selected populations that exhibit multiple resistance mechanisms including changes in tubulin isotype expression and mutations in β-tubulin [11], [12], [13], [14]. We have previously described epothilone B analogue resistant leukemia cells that exhibit multiple microtubule alterations including increased expression of βIII-tubulin, increased expression of MAP4, and mutations in βI-tubulin [13]. Whilst acquired resistance to epothilones has been described, research into intrinsic factors that mediate sensitivity to epothilones and related to the cellular target of the drug, tubulin, have been scarce. As these agents progress to the clinic it is important to understand how this class of compound interacts with different tubulin isotypes and how intrinsic levels of these proteins influence efficacy.

Using RNAi technology, we have previously shown that βIII-tubulin mediates sensitivity to paclitaxel and *Vinca* alkaloids in NSCLC cells [15]. Silencing the expression of βII- and βIVb-tubulin isotypes, on the other hand, enhance the sensitivity of these cells to *Vinca* alkaloids but not paclitaxel [16]. Correlative evidence that upregulation of βIII-tubulin does not mediate resistance to epothilone B has also been reported [12]. However, overexpression of βIII-tubulin in HeLa cells makes the cells less sensitive to epothilone B [17]. It is not known whether differential expression of β-tubulin isotypes influence response to epothilones. Understanding this interaction is highly desired for the development of predictive markers to provide more tailored therapy for NSCLC patients and other patients being treated with epothilones.

To investigate the functional significance of these β-tubulin isotypes in response to epothilone B in NSCLC, we employed RNAi technology to specifically knockdown the expression of these isotypes in two independent NSCLC cell lines and characterize the effects on cell morphology, sensitivity to epothilone B and drug-induced apoptosis.

## Materials and Methods

### Cell culture, siRNA transfection and cytotoxic drug

H460 and Calu-6 cells were obtained from ATCC (Manasses, VA, USA) and maintained as previously described [15]. Cell lines are routinely screened and free of mycoplasma. All transfection procedures were carried out as reported previously [15]. The potency and specificity of the siRNAs targeting each β-tubulin isotype have been validated previously [15], [16]. Epothilone B (Calbiochem, Merck biosciences) was prepared at a stock concentration of 100 µM in DMSO.

### Immunofluorescence staining

Briefly, siRNA-transfected Calu-6 cells growing in glass chamber slides were treated with epothilone B at the indicated concentrations for 1 h. Immunofluorescence staining of siRNA-transfected cells was then performed as previously described [15], [16].

### Drug-treated clonogenic assays

Drug-treated clonogenic assays were performed as previously described [15], [16]. The results were expressed as a surviving fraction and inhibitory dose (ID_50_) was extrapolated from the dose-response curve using GraphPad Prism program [15], [16].

### Cell cycle analysis

For analysis of DNA content by propidium iodide staining, H460 and Calu-6 cells were seeded in 6-well plates containing 6×10^4^ cells per well and transfected with siRNA. After 72 h transfection, cells were exposed to epothilone B at the indicated concentrations for 24 h. On the day of analysis, both adherent and floating cells were harvested, washed with PBS and fixed with 80% ethanol for at least 24 h at 4°C. The fixed cells were then stained with a solution containing 50 µg/ml propidium iodide, and 2 µg/ml DNase-free RNase for 30 min at 37°C in the dark. DNA content was measured by a FACSCalibur flow cytometer (BD). The CellQuest program was used to quantitate the distribution of cells in each cell cycle phase: sub-G_1_ (dead or fragmented), G_1_, S and G_2_-M [15], [16].

### Apoptosis assays

Cellular apoptosis was determined by measurement of caspase 3/7 activity using the Caspase-Glo 3/7 assay as previously described with slight modifications [18], [19]. Briefly, cells were transfected with siRNA for 24 h and replated in 96-well plates (5×10^3^ cells/well) and allowed to adhere for an additional 24 h. Cells were then treated with varying concentrations of epothilone for 24 h. Following treatment, cells were incubated with Caspase-Glo 3/7 reagent for 2 h at room temperature, and the luminescence was measured with a luminometer (PerkinElmer Victor 3). Additionally, apoptosis was also determined by Annexin V-FITC staining kit (Becton Dickinson) as previously described [15], [19].

### Statistical analysis

Data are expressed as the mean ± SEM and analyzed using ANOVA or student's *t* test followed by the nonparametric Dunnett or Mann-Whitney tests using the GraphPad Prism program. A P value of less than 0.05 was considered statistically significant.

## Results

### Differential sensitivity to epothilone B following βII-, βIII- or βIVb-tubulin knockdown

**Figure 1:**
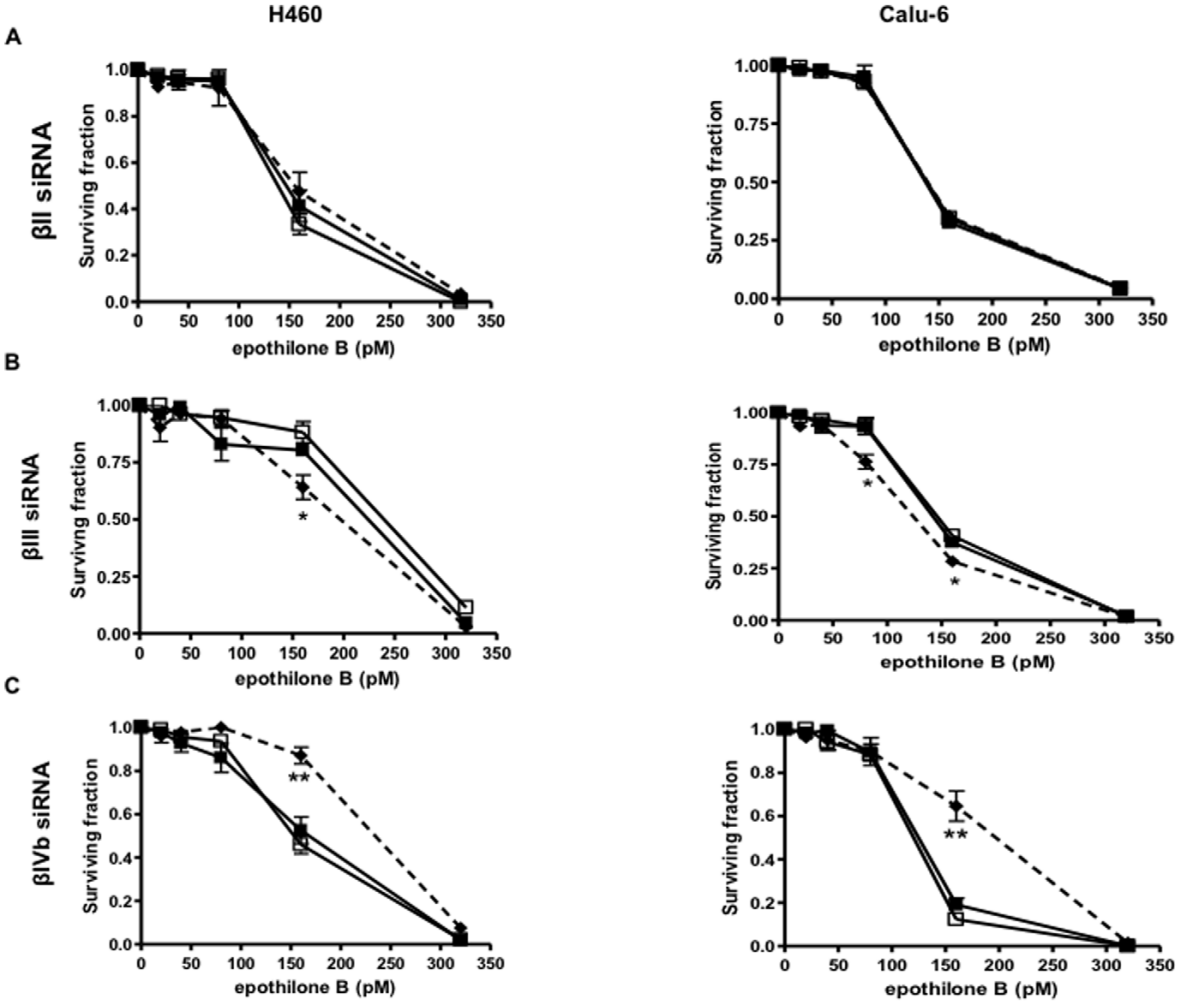
Drug-treated clonogenic assay. Clonogenic assays were performed on mock (closed squares, solid line), control siRNA (open squares, solid line) and specific β-tubulin isotype siRNA-transfected cells (closed diamonds, broken line) in two NSCLC cell lines, H460 (left panel) and Calu-6 (right panel). The graphs show the clonogenic survival of (A) βII-tubulin; (B) βIII-tubulin and (C) βIVb-tubulin knockdown cells exposed to epothilone expressed as surviving fraction. Bars, mean ± SEM of at least four individual assays. Statistics were calculated by comparing the surviving fraction of the knockdown cells with the mock-transfected cells at each drug concentration. *P<0.05, **P<0.01.

The specificity of each of the β-tubulin siRNA was confirmed at the protein level (Figure S1). Consistent with our previous studies, βII-, βIII-, and βIVb-tubulin siRNA potently inhibited protein expression of each of these targets respectively without affecting the expression of other major β-tubulin isotypes in the NSCLC cell lines (Figure S1) [15], [16]. To investigate the effects of these β-tubulin isotypes in response to epothilone B in NSCLC cells and to quantitate any changes in drug sensitivity, drug-treated clonogenic assays were performed. Knockdown of βII-tubulin expression in both H460 and Calu-6 cells did not affect sensitivity to epothilone B (Fig. 1A). In contrast, knockdown of βIII-tubulin significantly sensitized both NSCLC cell lines to epothilone B (Fig. 1B). Recently, we described the development and characterization of H460 cells selected for stable expression of shRNA against βIII-tubulin, and the increased sensitivity of these cells to paclitaxel, cisplatin and its analogue carboplatin [19]. Importantly, the increased sensitivity to epothilone B using transient knockdown of βIII-tubulin was also confirmed using the stable H460 βIII-tubulin shRNA knockdown cells (Figure S2), further strengthening our findings with this isotype. Interestingly, knockdown of βIVb-tubulin significantly reduced sensitivity to epothilone B in both cell lines, compared to mock- and control siRNA-transfected cells (Fig. 1C), suggesting that tumors expressing high levels of this isotype may be more sensitive to epothilone B than tumors with low levels of this isotype.

We also examined the differential effects of epothilone B on microtubules and cell morphology in βII-, βIII- and βIVb-tubulin knockdown cells. As shown in Figure S3, all untreated siRNA-transfected cells showed no obvious changes to microtubule structures, in concordance with our previous studies [15], [16]. However, epothilone B (5 nM) had a marked effect on cells with βIII-tubulin depleted microtubules. Microtubule bundles were more prominent in the βIII-tubulin knockdown cells. In contrast, microtubule networks remained largely organized and intact in control siRNA, βII- and βIVb-tubulin knockdown cells treated at the same concentration. At 20 nM epothilone B, both control and βII-tubulin knockdown cells start to exhibit microtubule bundles compared with the βIVb-tubulin knockdowns. These findings complement the clonogenic data and suggest that cells responded differently to epothilone B after specific knockdown of each individual β-tubulin isotype.

### Knockdown of βIII-tubulin reduces epothilone B induced cell cycle arrest and enhances cell death

**Figure 2:**
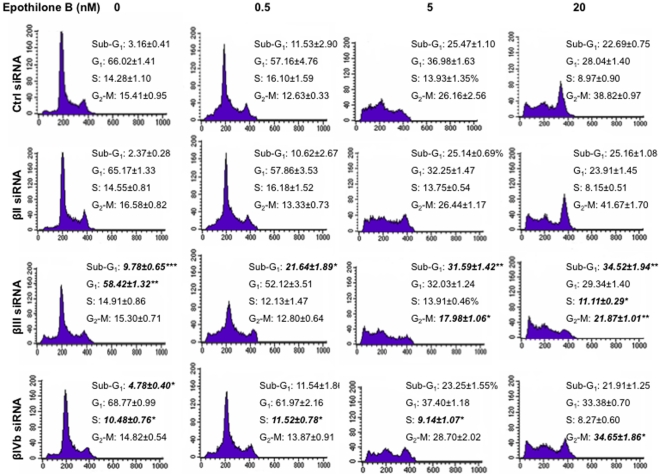
Cell cycle analysis of H460 knockdown cells treated with epothilone B. Drug concentrations were indicated on top of the figure. Cells were harvested after 24 h drug treatment and subsequently assayed for their DNA content by flow cytometry as described in Materials and Methods. Representative figures of four independent experiments are shown. *P<0.05, **P<0.01.

Cell cycle analysis was performed next to determine whether knockdown of each β-tubulin isotype influences cell cycle profiles in the presence of epothilone B for 24 h. When treated with concentrations as low as 0.5 nM epothilone B, the βIII-tubulin knockdown cells showed a significant increase in sub-G_1_ content, indicative of cell death (Fig. 2). A greater difference was observed with 20 nM epothilone B, with the control siRNA- and βII-tubulin siRNA-treated cells showing a marked G_2_-M block whereas the βIII-tubulin knockdown cells displayed an increase in the sub-G_1_ population (Fig. 2). βIII-tubulin knockdown cells had less cells accumulating at G2-M compared to controls, suggesting that cell death may be occurring independent of mitotic arrest. It is evident that knockdown of βIII-tubulin strongly increases sensitivity to epothilone B via increased cell death because the sub-G_1_ population was increased at all concentrations tested. In βIVb-tubulin knockdown cells on the other hand, a lower G_2_-M content was observed when compared with the control siRNA-treated cells at 20 nM epothilone B (Fig. 2). The sub-G_1_ content did not differ between βIVb-tubulin knockdown and the control siRNA-treated cells at 20 nM. In contrast, knockdown of βIVb-tubulin, showed a lower number of cells blocked at G_2_-M, thereby confirming the decrease in sensitivity of these cells to epothilone B.

To determine whether epothilone B-induced G_2_-M cell cycle delay was occurring at earlier time points, cell cycle analysis using H460 and Calu-6 cells was performed at 4, 8 and 12 h in the presence or absence of epothilone B (20 nM). In the presence of the drug, G_2_-M cell cycle arrest was observed as early as 4 h for H460 cells (Table S1) and 8 h for Calu-6 cells (Table S2) in both βIII-tubulin knockdown and control-siRNA transfected cells. At 8 and 12 h the percentage of H460 cells blocked at G_2_-M was lower than control (Table S1), although a similar trend was not observed in the Calu-6 cells (Table S2).

### Sensitivity to epothilone B correlates with the level of apoptosis induction

**Figure 3:**
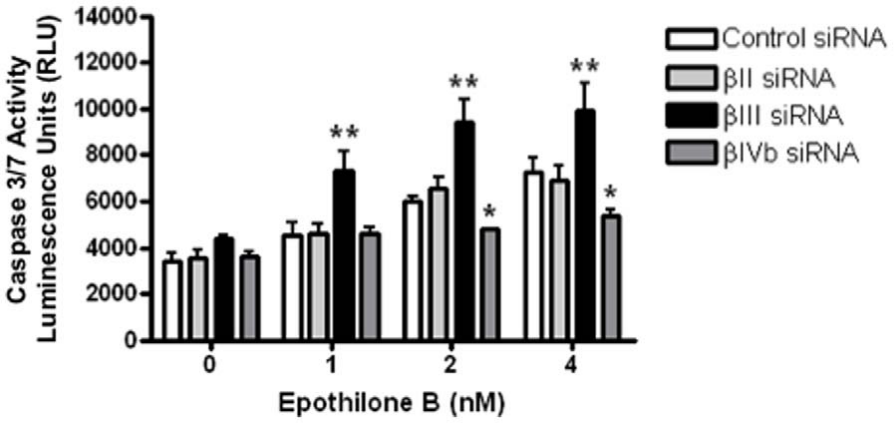
Caspase activity 3/7 assay. siRNA-transfected H460 cells were harvested after 24 h incubation in the presence or absence of epothilone B and subsequently assayed for apoptosis induction by caspase activity assay. Open bars: control siRNA-transfected cells; light grey solid bars: βII-tubulin knockdown cells; solid black bars: βIII-tubulin knockdown cells; dark grey solid bars: βIVb-tubulin knockdown cells. Data represent means ± SEM of at least three independent experiments. *P<0.05; **P<0.01.

To address whether the increased or decreased sensitivity to epothilone B specific to each β-tubulin isotype was related to apoptosis induction, we measured caspase 3/7 activity in these cells after 24 h drug treatment. Caspase 3/7 activity in the βIII-tubulin knockdown cells was increased at least 2-fold over that in the control siRNA-transfected cells at all concentrations tested (Fig. 3). The increased caspase activity in the βIII-tubulin knockdown cells correlated with the increased cell death observed in these cells upon drug treatment. In contrast, caspase 3/7 activity remained at background levels in βII- and βIVb-tubulin knockdown cells, similar to the control siRNA cells at ≤1 nM epothilone B. Importantly, there was a significant decrease in caspase activity in the βIVb knockdown cells at ≥2 nM, suggesting the βIVb-tubulin knockdowns were less sensitive to epothilone-induced apoptosis.

**Figure 4:**
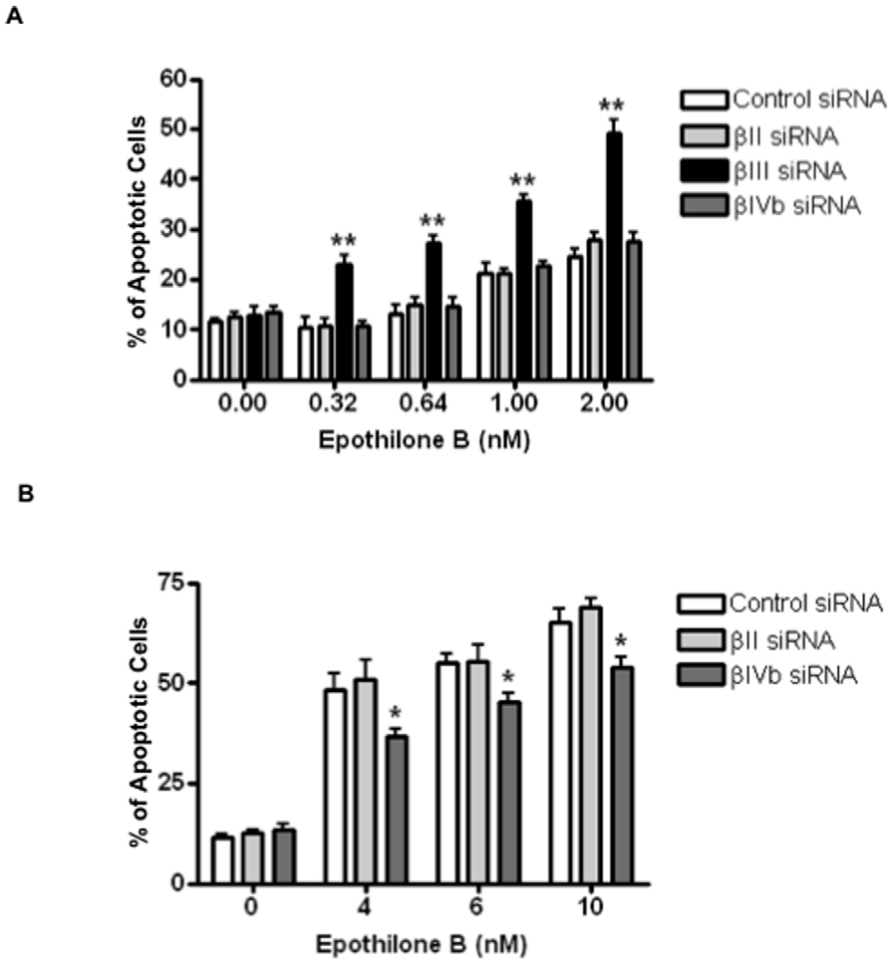
Annexin V staining of siRNA-transfected H460 cells following 48 h incubation with epothilone B. Open bars: control siRNA-transfected cells; light grey solid bars: βII-tubulin knockdown cells; solid black bars: βIII-tubulin knockdown cells; dark grey solid bars: βIVb-tubulin knockdown cells. Note epothilone B was able to induce apoptosis in the βIII-tubulin knockodown cells at concentrations as low as 0.32 pM (A), whereas higher concentrations of epothilone B induce significantly lower apoptosis in the βIVb-tubulin knockdown cells (B). Data represent means ± SEM of at least three independent experiments. *P<0.05; **P<0.01.

To further define the role of β-tubulin isotypes in epothilone B-induced apoptosis, Annexin V-FITC staining was also performed following 48 h treatment with epothilone B. As shown in Fig. 4A, treatment of βIII-tubulin knockdown H460 cells induced apoptosis from a concentration as low as 320 pM of epothilone B. The percentage of apoptotic cells was significantly higher in the βIII-tubulin siRNA-treated cells than in control, βII- or βIVb-tubulin siRNA-treated cells at all concentrations tested (Fig. 4A). In contrast, a higher concentration of epothilone B was needed to induce apoptosis in βIVb-tubulin knockdown cells compared to either control or βII-tubulin siRNA-transfected cells (Fig. 4B). Taken together, this data shows that increased apoptosis induction might be one of the mechanisms underlying the hypersensitivity to epothilone B following βIII-tubulin knockdown. Knockdown of βIVb-tubulin, on the other hand, decreased sensitivity to epothilone B-induced apoptosis induction in NSCLC.

## Discussion

The epothilones represent a novel class of microtubule stabilizing agents that could potentially provide another approach to overcome paclitaxel resistance. Epothilones have shown promising clinical activity in a phase II trial in NSCLC patients [20] and have been recently approved for use in metastatic breast cancer. It is not known how this class of compound interacts with different tubulin isotypes and how these influence epothilone B sensitivity. Here in, we show that specific β-tubulin isotypes differentially affect NSCLC cell sensitivity to epothilones (Table 1).

**Table 1:** Summary of the differential response of β-tubulin isotypes to epothilone B.

β-tubulin isotype	Epothilone B sensitivity	G_2_/M cell cycle arrest	Apoptosis induction*
βII-tubulin siRNA	No change	Yes	Same as control siRNA cells at all concentrations tested
βIII-tubulin siRNA	Increased	Decreased	Increased with epothilone B treatment at all concentrations tested
βIVb-tubulin siRNA	Decreased	Yes	Same as control siRNA at low concentrations then decreased at higher concentrations (≥2 nM)

*Apoptosis induction was measured by caspase activity assay and Annexin V staining.

Although altered expression of β-tubulin isotypes has been associated extensively with taxane resistance, limited information is available on the role of β-tubulin isotypes in sensitivity to epothilones. In this study, βII-tubulin did not affect sensitivity to epothilone B in either of the two independent NSCLC cell lines examined, H460 and Calu-6 cells. Interestingly, sensitivity to the microtubule stabilizing agent paclitaxel does not appear to be influenced by overexpression or suppression of βII-tubulin expression [16], [21]. This result contrasts with our previous study examining *vinca* alkaloids, where suppression of βII-tubulin enhances sensitivity to these agents [16].

Preclinical and clinical studies have previously shown that drug resistance to TBAs is often associated with βIII-tubulin upregulation (Reviewed in [10], [22]). There has been speculation and correlative evidence that the cytotoxic effects of epothilones are independent of βIII-tubulin expression because of their activity in βIII-tubulin overexpressing cells *in vitro* and in human xenograft models [9], [12]. However, definitive evidence has not been shown that epothilone activity is truly independent of βIII-tubulin expression. Using RNAi technology, we show that knockdown of βIII-tubulin leads to a significant increase in sensitivity to epothilone B. In agreement, stable overexpression of βIII-tubulin in HeLa cells was found to confer resistance to a range of TBAs including epothilone B [17]. One report described epothilone-resistant ovarian cancer cell lines with decreased βIII-tubulin expression [12]. Drug resistance is multifactorial and different cell line models could account for differences. Importantly, other changes in β-tubulin isotypes and βI-tubulin point mutations were also observed in the epothilone B-resistant cell lines [12], suggesting that these factors might have also contributed to the resistant phenotype. We have previously described epothilone B analog resistant leukaemia cells that displayed multiple microtubule alterations including increased expression of βIII-tubulin expression and βI-tubulin mutations [13]. To date, the contributions of acquired epothilone B resistance mechanisms have not been well correlated with intrinsic sensitivity to epothilones. It should be stressed that the two independent NSCLC cells used in the current study have neither been subjected to prior drug selection nor express P-glycoprotein (data not shown) and therefore provide an opportunity for assessing sensitivity to epothilone B conferred by each of the β-tubulin isotypes examined.

**Figure 5:**
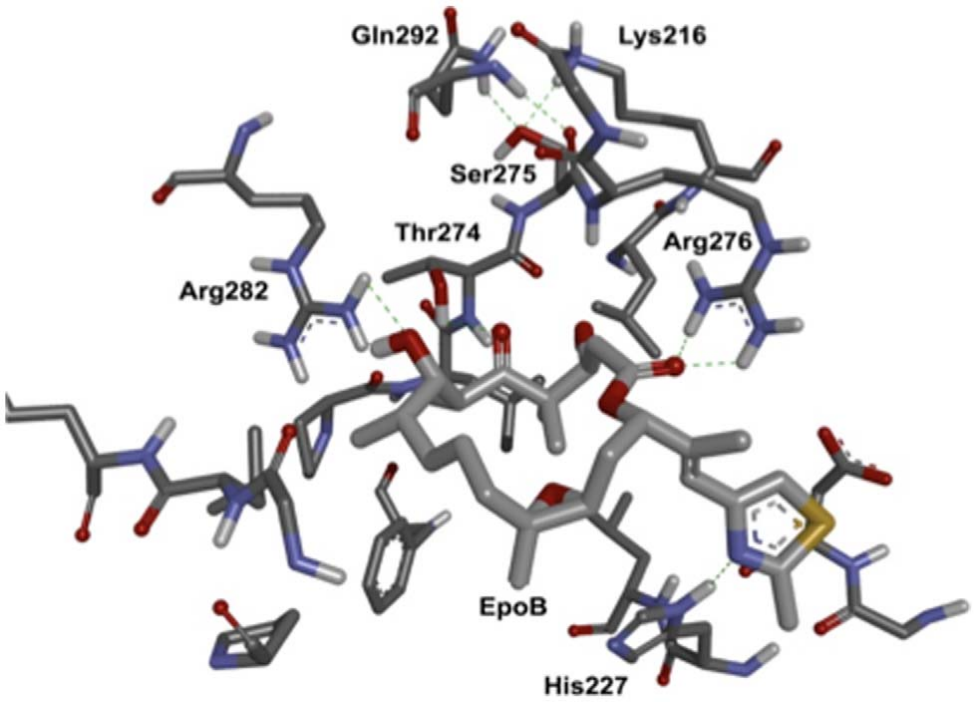
The putative binding pocket (all residues within 6 Å of the ligand) of tubulin (1TVK), with epothilone B (modified from epothilone A in 1TVK). Epothilone B is shown as sticks (light grey carbons). The binding pocket residues of 1TVK are shown as sticks (dark grey carbons). Non-polar hydrogens are omitted for clarity. Hydrogen bonds are shown as dashed green lines. Ser275 can form 3 hydrogen bonds with Gln292 and Lys216. Images generated in DS Modelling 3.0 (Accelrys®).

Interestingly, while knocking down βIII-tubulin hypersensitizes the cells to epothilone B, knockdown of βIVb-tubulin decreased the sensitivity of the NSCLC cells to epothilone B. Recently, Cabral and co-workers have reported that cells overexpressing βIVb-tubulin exhibited a small but significant increase in sensitivity to epothilone A [23]. Taken together with our study, βIVb-tubulin expression may be a favourable therapeutic indicator for epothilone B therapy. We have previously shown that knockdown of βII- and βIVb-tubulins in the NSCLC cells used in this study did not significantly affect paclitaxel sensitivity, but did significantly increase sensitivity to vinca alkaloids [16]. Hence, despite paclitaxel and epothilone B sharing overlapping binding sites on β-tubulin, βIVb-tubulin expression levels elicit distinct effects on sensitivity to paclitaxel and epothilone B. There is growing evidence showing that the binding of epothilones and paclitaxel to tubulin may not be identical [13], [24]. Evidently, some point mutations in the β-tubulin subunit confer paclitaxel but not epothilone resistance in cell culture models [7], [25], suggesting that epothilones and taxanes may have distinct interactions with β-tubulin isotypes. A rationalisation for the differences in sensitivity induced by β-tubulin isotype expression may be related to amino acid differences between the isotypes. Beta-tubulin isotypes βI, βII, βIVa and βIVb, share at least 95% identity and have a limited number of non-conservative amino acid substitutions (Figure S4) [26]. In contrast, βIII-tubulin differs as it shares only 92% identity to the above β-tubulin isotypes. Within the paclitaxel/epothilone binding pocket, in βII and βIVb isotypes, Ser275 has been implicated as a mediator of paclitaxel diffusion through nanopores [27] and can hydrogen bond with Gln279 and Lys216, stabilising the M-loop (Fig. 5). In turn, this may enhance the hydrogen bonds that are observed between Arg276 to the lactone carbonyl and Thr274 to the ketone oxygen on C5 of epothilone A (and presumably epothilone B). In βIII-tubulin, there is a Ser275Ala mutation that could destabilise the M-loop and thereby weaken the Arg226 and Thr274 hydrogen bonds with the ligand, contributing to its reduced sensitivity. However, this does not explain the differential sensitivity observed between βIII-tubulin and βIVb, as the amino acid sequences identified as being important within the paclitaxel/epothilone binding pocket, or the GDP binding site do not differ between these two isotypes [24]. The current study cannot exclude the possibility that the differential expression of specific β-tubulin isotypes affects the binding of epothilones to the microtubule wall, or through stabilisation of contacts between dimers in forming protofilaments. However, a recent study with epothilone A showed it binds equally well to both βI- and βIII-tubulin [28]. A similar study examining the binding of epothilone B and specific β-tubulin isotypes would be important to determine how these isotypes affect the interaction of this drug with tubulin.

Antitumour activity of epothilones is mediated by suppression of microtubule dynamics, mitotic arrest at the G_2_-M cell cycle phase followed by apoptosis. To address the potential mechanisms underlying the differential response to epothilone B following knockdown of a specific β-tubulin isotype, we examined the propensity of the cells to undergo drug-induced cell cycle arrest and apoptosis. Following incubation with epothilone B, βIII-tubulin knockdown showed an increase in the sub-G_1_ populations (cell death) whilst a decrease in G_2_-M block when compared to the control siRNA-transfected cells. Knockdown of βIII-tubulin can significantly reduce the extent of mitotic block induced by incubation with either paclitaxel or vincristine [15], whilst increasing the level of cell death. The effect on epothilone B sensitivity cannot be simply explained by a change in microtubule dynamics, as we recently demonstrated that microtubule dynamics do not change in H460 cells following βIII-tubulin knockdown [29]. Collectively, these studies demonstrate that knockdown of βIII-tubulin may enhance TBA-induced apoptotic cell death via a separate pathway that is independent of mitotic arrest. Another study has shown that the anti-tumour effects of paclitaxel, correlated with paclitaxel-induced apoptosis but not with mitotic arrest [30]. Epothilones might have a similar mechanism of action. Interestingly, βIVb-tubulin knockdown cells had a decrease in the number of cells blocked at G_2_-M (epothilone B 20 nM) as compared to control and βII-tubulin knockdown cells, albeit at a level higher than the βIII-tubulin knockdown cells. However, unlike βIII-tubulin knockdown cells, βIVb-tubulin knockdown cells undergo drug-induced cell death at a similar level as the control and βII-tubulin knockdown cells. Further, both the caspase 3/7 activity and Annexin V staining showed that βIII-tubulin knockdown cells had a significant increase in epothilone B-induced apoptosis induction at all concentrations tested. In contrast, knockdown of βIVb-tubulin protected cells against epothilone B as reflected in decreased induction of apoptosis. Hence, apoptosis induction might serve as one of the mechanisms underlying the increased or decreased sensitivity observed with these specific β-tubulin isotypes in response to epothilone B.

The molecular link between β-tubulin and epothilone B-induced apoptosis remains to be established. It has been shown recently that epothilone B induced apoptosis in human neuroblastoma cells by increasing the generation of reactive oxygen species from mitochondria and subsequently relocalization of the proapoptotic protein Bim in the mitochondria compartment [31]. Future investigations will determine whether ROS generation and mitochondria or expression of different pro- and antiapoptotic proteins are responsible for the ability of βIII-tubulin or βIVb-tubulin to differentially affect epothilone B-induced apoptotic signals, and whether these signals occur independent of mitotic arrest.

The significance of differential β-tubulin isotypes in sensitivity to epothilone B requires further validation in the clinical setting to assess its applicability in predicting the efficacy of epothilone B. It will also be of great interest to determine whether expression of βIVb-tubulin will correlate with clinical response in cancers treated with epothilone, as based on the results in this study, tumours with high βIVb-tubulin levels would be expected to be more sensitive to this agent.

Taken together, these results show that β-tubulin isotype composition of a cell affects sensitivity to epothilone B. Clinical studies are warranted to assess the therapeutic value of differential expression of β-tubulin isotypes in NSCLC and their role in clinical response to epothilones.

## Supporting Information

Figure S1 **siRNA targeting βII, βIII or βIVb-tubulin specifically silences their expression in H460 and Calu-6 NSCLC cells**. Representative western blots showing siRNA targeting βII (**A**), βIII (**B**), or βIVb-tubulin (**C**) inhibits its protein expression in H460 and Calu-6 NSCLC cells when compared to cells treated with control siRNA or Mock (lipofectamine 2000 only). No significant changes in the expression of other β-tubulin isotypes were observed. Glyceraldehyde-3-phosphate dehydrogenase (*GAPDH*) expression was used as a loading control. Representative gels. n =  3 separate experiments.
(TIFF)

Figure S2 **Stable and potent inhibition of βIII-tubuin increases sensitivity to epothilone B in H460 NSCLC cells.** Clonogenic assay showing the effect of stable knockdown of βIII-tubulin on sensitivity to epthoilone B in H460 cells expressing shRNA targeting βIII-tubulin (pRS/βIII_SH4_) (dashed line) or control (pRS/Ctrl_SH2_) (solid line). *Points*, means; *bars* SE (n = 6 individual experiments,*p<0.01).
(TIFF)

Figure S3 **Effect of βII-, βIII- and βIVb-tubulin knockdown on the microtubule network.** Calu-6 transfected cells were fixed and stained with an antibody to α-tubulin after 72 h transfection. Arrows represent dying cells. Scale bar-20 µm.
(TIFF)

Figure S4 Sequence alignment of the β-subunit of 1TVK with the sequences of βIIb, βIII and βIVb tubulin. Identical sequences are shaded grey, strong matching (dark blue), weak matching (light blue) and non matching residues are unshaded. The residues of the epothilone binding pocket (within 6 Å of the ligand) are highlighted in black.
(TIFF)

Table S1 (DOCX)

Table S2 (DOCX)
