# Supplementary figures and images for: Specific β-Tubulin Isotypes Can Functionally Enhance or Diminish Epothilone B Sensitivity in Non-Small Cell Lung Cancer Cells
Source: PLoS One. 2011 Jun 29;6(6):e21717. doi: 10.1371/journal.pone.0021717 (PMC3126859; doi:10.1371/journal.pone.0021717)

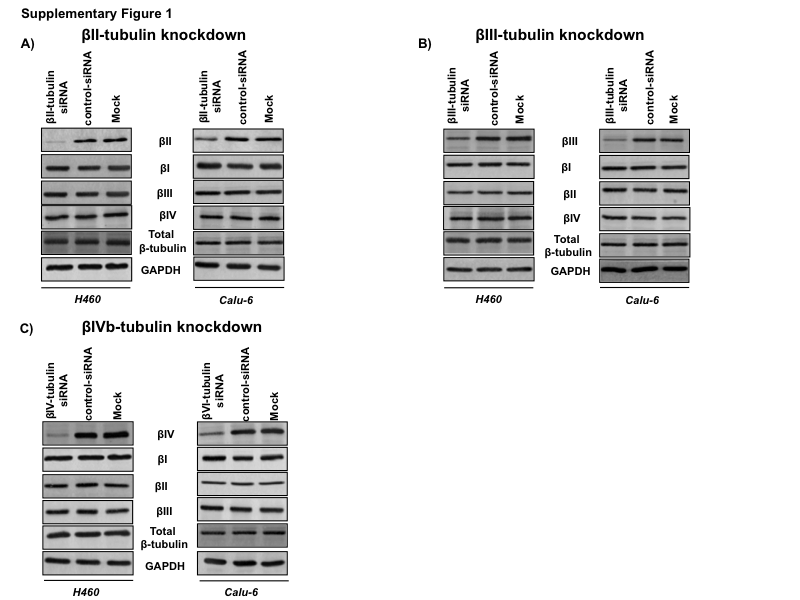

Supplement: Figure S1 — siRNA targeting βII, βIII or βIVb-tubulin specifically silences their expression in H460 and Calu-6 NSCLC cells. Representative western blots showing siRNA targeting βII (A), βIII (B), or βIVb-tubulin (C) inhibits its protein expression in H460 and Calu-6 NSCLC cells when compared to cells treated with control siRNA or Mock (lipofectamine 2000 only). No significant changes in the expression of other β-tubulin isotypes were observed. Glyceraldehyde-3-phosphate dehydrogenase (GAPDH) expression was used as a loading control. Representative gels. n = 3 separate experiments. (TIFF) [file pone.0021717.s001.tiff]

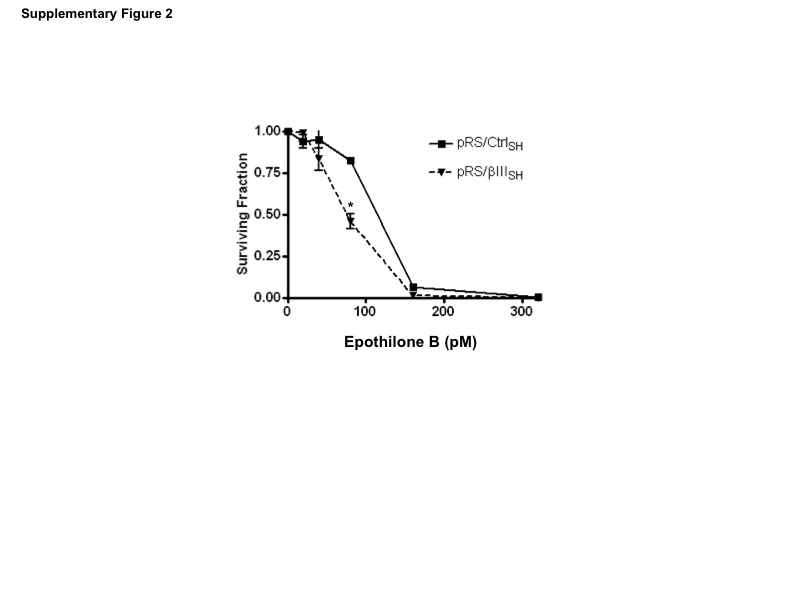

Supplement: Figure S2 — Stable and potent inhibition of βIII-tubuin increases sensitivity to epothilone B in H460 NSCLC cells. Clonogenic assay showing the effect of stable knockdown of βIII-tubulin on sensitivity to epthoilone B in H460 cells expressing shRNA targeting βIII-tubulin (pRS/βIIISH4) (dashed line) or control (pRS/CtrlSH2) (solid line). Points, means; bars SE (n = 6 individual experiments,*p<0.01). (TIFF) [file pone.0021717.s002.tiff]

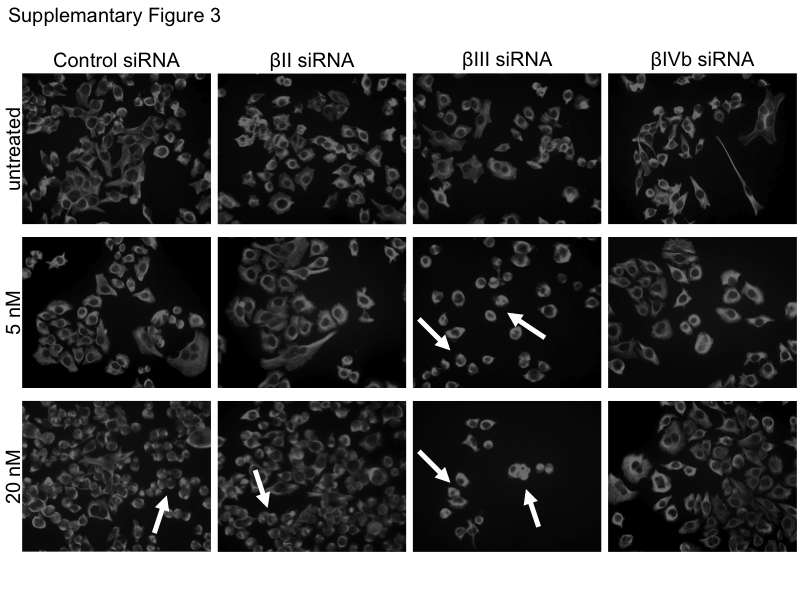

Supplement: Figure S3 — Effect of βII-, βIII- and βIVb-tubulin knockdown on the microtubule network. Calu-6 transfected cells were fixed and stained with an antibody to α-tubulin after 72 h transfection. Arrows represent dying cells. Scale bar-20 µm. (TIFF) [file pone.0021717.s003.tiff]

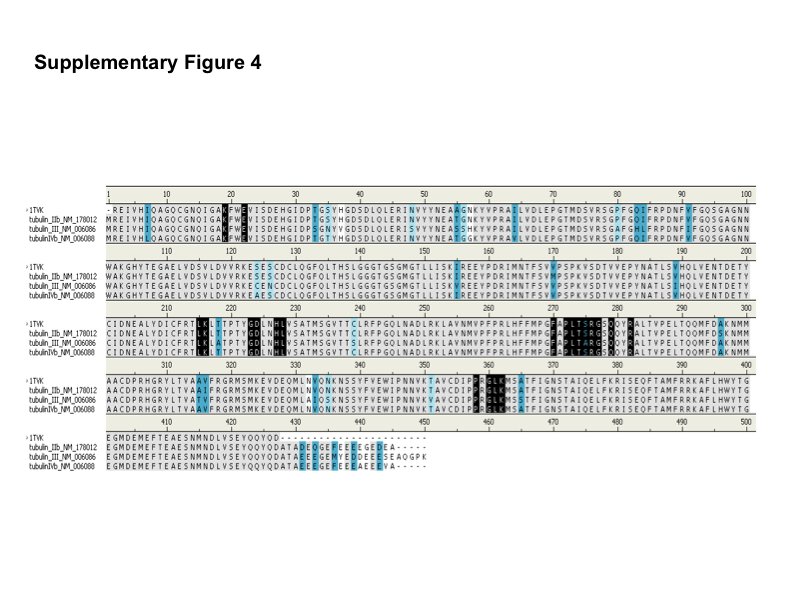

Supplement: Figure S4 — Sequence alignment of the β-subunit of 1TVK with the sequences of βIIb, βIII and βIVb tubulin. Identical sequences are shaded grey, strong matching (dark blue), weak matching (light blue) and non matching residues are unshaded. The residues of the epothilone binding pocket (within 6 Å of the ligand) are highlighted in black. (TIFF) [file pone.0021717.s004.tiff]
