# Supplementary material for: Specific β-Tubulin Isotypes Can Functionally Enhance or Diminish Epothilone B Sensitivity in Non-Small Cell Lung Cancer Cells
Source: PLoS One. 2011 Jun 29;6(6):e21717. doi: 10.1371/journal.pone.0021717 (PMC3126859; doi:10.1371/journal.pone.0021717)
Supplement: Table S1 — (DOCX) [file pone.0021717.s005.docx]

**Supplementary Table 1. Effect of epothilone B on cell cycle distribution of the βIII-tubulin and control siRNA transfected H460 cells#.**

| **Cell cycle phases** | **Time**  **(h)** | **Control siRNA** | | **βIII-tubulin siRNA** | |
| --- | --- | --- | --- | --- | --- |
|  |  | Untreated | 20nM EpoB | Untreated | 20nM EpoB |
| Sub G_1_ | 4 | 1.54±0.24 | 2.51±0.07 | ***7.97±2.24**** | ***8.12±1.07***** |
|  | 8 | 2.07±0.38 | 8.01±1.74 | ***7.33±1.39**** | ***19.86±3.96****** |
|  | 12 | 1.65±0.55 | 18.22±1.46 | 6.31±1.87 | ***32.56±1.99***** |
| G_0_/G_1_ | 4 | 67.97±1.25 | 49.25±2.02 | ***60.11±1.24***** | ***42.53±1.07**** |
|  | 8 | 68.29±1.41 | 20.25±1.37 | 60.81±2.89 | 17.01±0.57 |
|  | 12 | 67.91±0.91 | 15.0±1.61 | ***60.24±0.85***** | 13.2±0.49 |
| S | 4 | 15.01±1.3 | 18.04±1.27 | 16.61±1.59 | 20.94±0.85 |
|  | 8 | 15.76±1.54 | 20.24±0.55 | 17.3±1.48 | 20.85±0.87 |
|  | 12 | 16.93±1.36 | 8.69±0.78 | 18.97±0.83 | 10.39±1.95 |
| G_2_/M | 4 | 15.54±0.26 | 30.4±1.71 | 15.58±1.04 | 28.69±0.95 |
|  | 8 | 14.32±0.62 | 51.94±1.83 | 15.8±0.44 | 43.02±5.03 |
|  | 12 | 13.92±1.21 | 58.33±1.17 | 15.12±2.64 | ***44.11±3.27***** |

#H460 cells were transfected with 5nmol/L control siRNA (Qiagen) or βIII-tubulin siRNA (Dharmacon). After 72 hours, cells were exposed to 20nmol/L Epothilone B for the indicated times (4-12 hours). Cells were harvested and stained with propidium iodide and analysed by flow cytometry.

Values are means % ± SEM of three independent experiments. Values significantly different from control siRNA-treated cells (untreated or Epothilone B treated) are in bold/italic; * p<0.5 ** p<0.05.
