# Supplementary material for: Specific β-Tubulin Isotypes Can Functionally Enhance or Diminish Epothilone B Sensitivity in Non-Small Cell Lung Cancer Cells
Source: PLoS One. 2011 Jun 29;6(6):e21717. doi: 10.1371/journal.pone.0021717 (PMC3126859; doi:10.1371/journal.pone.0021717)
Supplement: Table S2 — (DOCX) [file pone.0021717.s006.docx]

**Supplementary Table 2. Effect of epothilone B on cell cycle distribution of the βIII-tubulin and control siRNA transfected Calu-6 cells#**

| **Cell cycle phases** | **Time**  **(h)** | **Control siRNA** | | **βIII-tubulin siRNA** | |
| --- | --- | --- | --- | --- | --- |
|  |  | Untreated | 20nM EpoB | Untreated | 20nM EpoB |
| Sub G_1_ | 4 | 18.94±1.32 | 16.27±1.29 | 17.62±1.19 | 15.4±0.67 |
|  | 8 | 19.28±0.61 | 12.47±1.51 | 18.3±1.27 | 13.14±1.04 |
|  | 12 | 20.47±1.47 | 11.74±1.6 | 19.28±0.93 | 10.53±1.73 |
| G_0_/G_1_ | 4 | 47.35±1.34 | 46.8±1.28 | 46.12±0.73 | 45.47±0.83 |
|  | 8 | 48.21±1.43 | 40.2±2.23 | 48.09±0.1 | 40.08±1.95 |
|  | 12 | 50.87±0.8 | 32.81±1.85 | 50.43±1.48 | 33.29±1.46 |
| S | 4 | 13.26±1.24 | 13.47±0.97 | 14.86±0.72 | 15.81±0.78 |
|  | 8 | 12.48±0.63 | 14.46±1.11 | 13.73±0.27 | 16.54±1.16 |
|  | 12 | 12.52±0.59 | 14.51±0.4 | 13.05±0.27 | 15.2±0.54 |
| G_2_/M | 4 | 20.76±1.86 | 23.79±1.88 | 21.74±1.17 | 23.73±0.91 |
|  | 8 | 20.27±1.4 | 33.31±2.8 | 20.31±1.21 | 30.68±1.93 |
|  | 12 | 16.47±0.63 | 41.14±0.28 | 17.57±0.88 | 41.13±3.25 |

#Calu-6 cells were transfected with 5nmol/L control siRNA (Qiagen) or βIII-tubulin siRNA (Dharmacon). After 72 hours, cells were exposed to 20nmol/L Epothilone B for the indicated times (4-12 hours). Cells were harvested and stained with propidium iodide and analysed by flow cytometry.

Values are means % ± SEM of three independent experiments.
